# Vesiculopustular drug reaction with eosinophilia and systemic symptoms induced by levetiracetam

**DOI:** 10.1002/ski2.384

**Published:** 2024-04-11

**Authors:** Thomas Norman, Jana Guenther, Kevin Wu, Brittney DeClerck, Scott Worswick

**Affiliations:** ^1^ Keck School of Medicine University of Southern California Los Angeles California USA; ^2^ Department of Dermatology Keck School of Medicine University of Southern California Los Angeles California USA

## Abstract

Drug reaction with eosinophilia and systemic symptoms (DRESS) is a severe cutaneous adverse reaction characterised by fever, lymphadenopathy, morbilliform rash, haematologic abnormalities, and multiorgan involvement. Herein, we describe a 32‐year‐old female presenting with a 9‐day history of facial oedema, cervical and inguinal lymphadenopathy, and a pruritic rash comprised of vesicles and pustules on her face, trunk, and extremities. Her only medications were valproate, which she had been taking for several years, and levetiracetam, which was initiated 41 days prior to rash onset. On the 16th day of her rash, she was diagnosed with DRESS induced by levetiracetam (Registry of Severe Cutaneous Adverse Reactions: 5). At this point, her absolute eosinophil count was 0.9 × 10^9^ cells/L and aspartate and alanine transaminase levels were 357 and 339 U/L, respectively. Pustules with a morbilliform rash may occur in up to 30% of DRESS cases. In rarer instances, as in our patient, DRESS can present with isolated pustules and vesicles. Similarly, although rare, DRESS can be induced by levetiracetam.

## Graphical abstract

We describe a 32‐year‐old‐female with a vesiculopustular variant DRESS induced by levetiracetam. Our case presents both a rare cutaneous morphology and an uncommonly implicated drug.

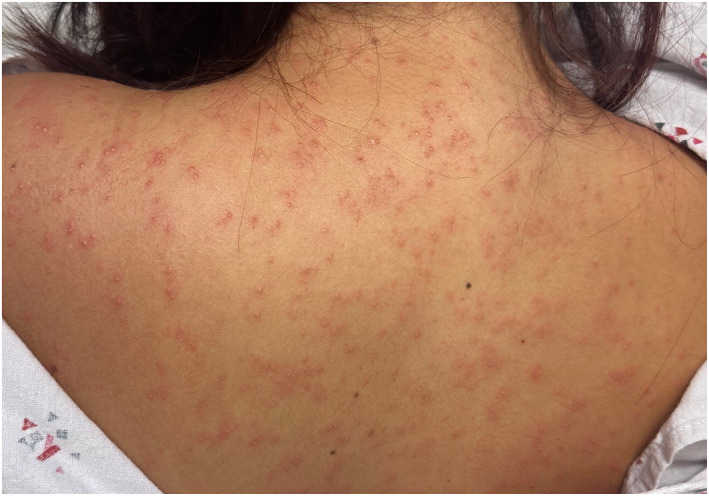

## 1 INTRODUCTION

Drug reaction with eosinophilia and systemic symptoms (DRESS) is a severe cutaneous adverse reaction developing 2 to 6 weeks after initiation of the culprit drug. Clinical hallmarks include fever, lymphadenopathy, morbilliform rash, haematologic abnormalities, and multiorgan involvement.^1^, ^2^ Given the potential mortality, early recognition is important to allow for discontinuation of the responsible drug and, if needed, initiation of immunomodulatory therapy.^2^ Herein, we present a case of vesiculopustular DRESS induced by levetiracetam, unique in both morphology and implicated drug.

## 2 CASE PRESENTATION

**FIGURE 1:**
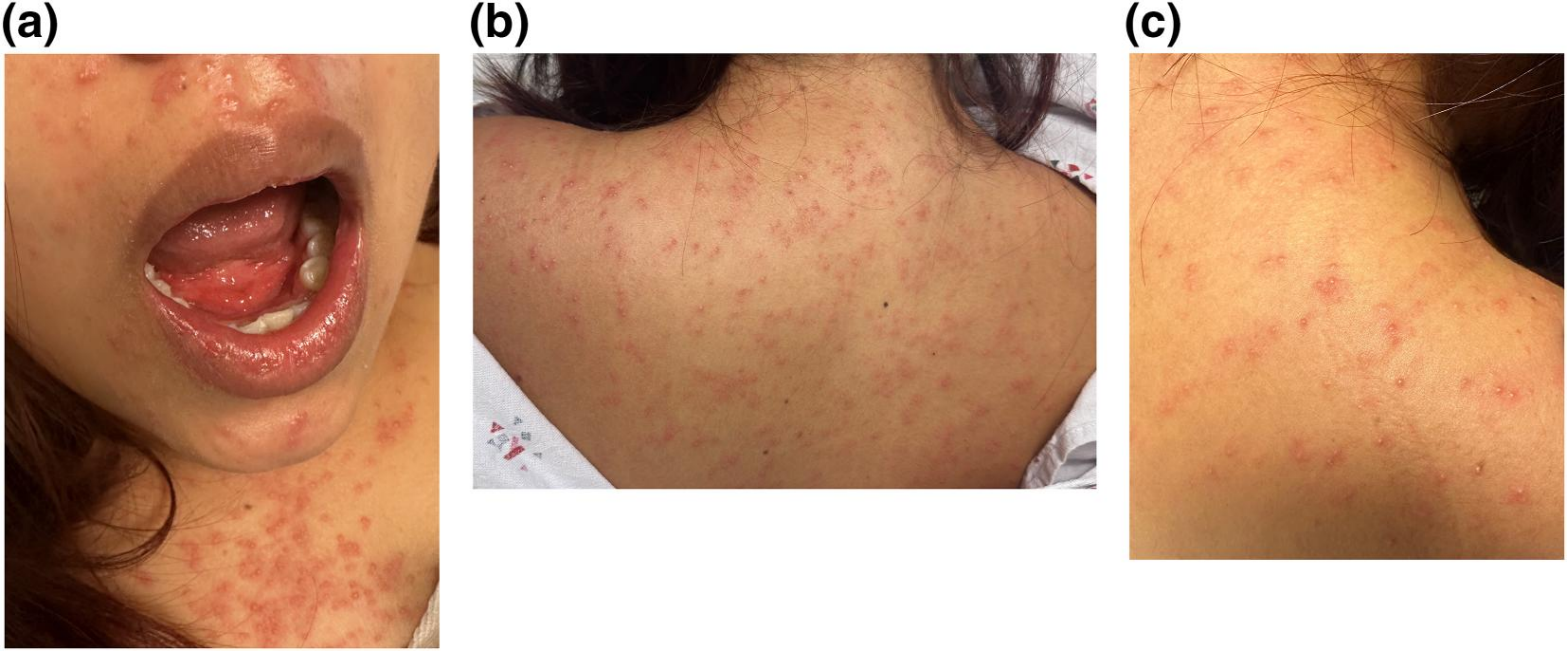
Vesicles and pustules with surrounding erythema on the face (a), upper chest (a), and upper back (b and c) at hospital admission on rash day 9.

A 32‐year‐old Hispanic female with epilepsy presented to the emergency department with a 9‐day history of a pruritic rash consisting of scattered vesicles and pustules (VP) with surrounding erythema that developed on her face before spreading to her trunk and extremities (Figure 1a–c). She denied sick contacts, joint pain, and recent weight loss. Her only medications were valproate 500 mg twice daily, which she had been taking for several years, and levetiracetam 500 mg twice daily, initiated 41 days prior to rash onset. She was admitted, and both anticonvulsants were discontinued.

**FIGURE 2:**
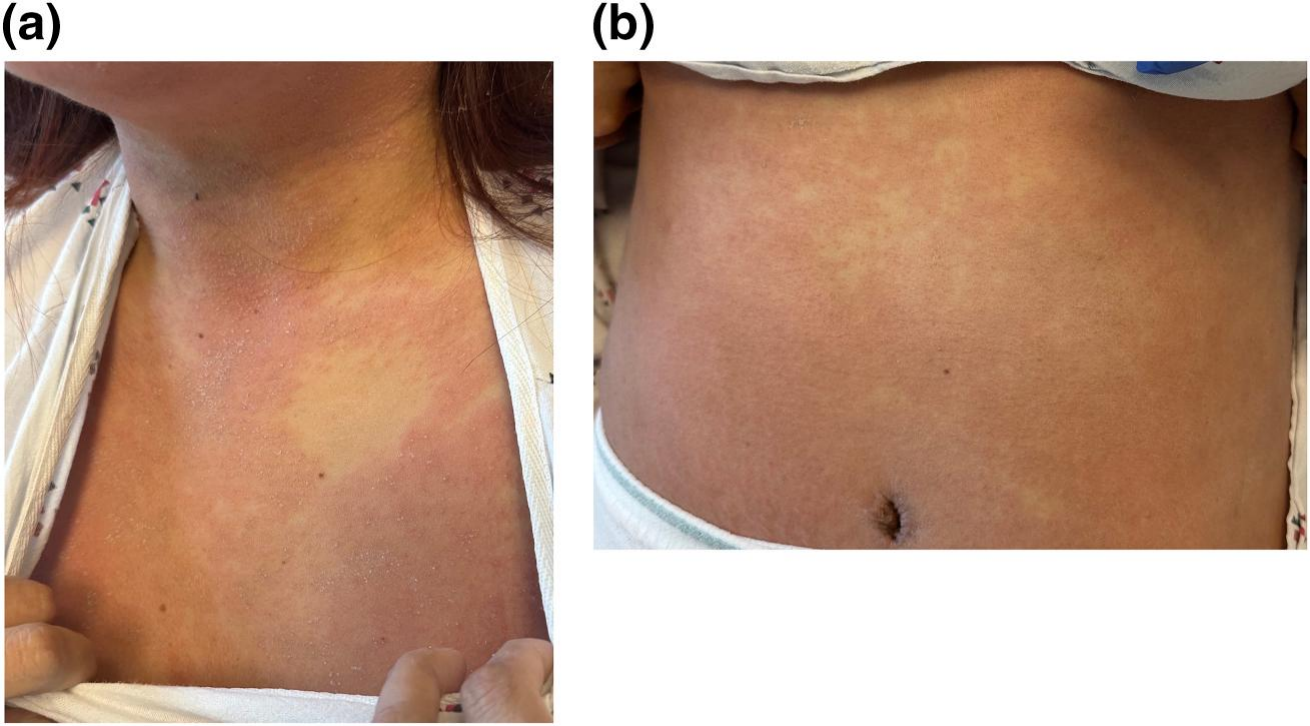
Confluent erythematous patches with islands of sparing on the upper chest (a) and lower abdomen (b) on rash day 16. A few scattered vesicles and pustules were still present. Overlying white flaking partly attributed to dried calamine lotion.

**FIGURE 3:**
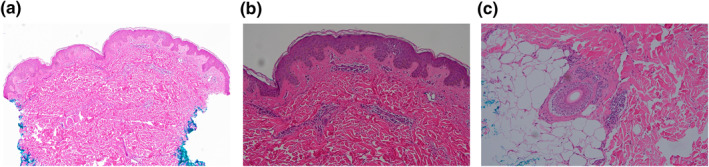
Haematoxylin and eosin stain of punch biopsy revealing very mild perivascular dermatitis with a focal, small aggregate of atypical hematolymphoid cells within the deep dermis. Magnifications: (a) 10×; (b) 20×; (c) 20×.

Throughout her hospitalisation, she was afebrile but had facial oedema and painful cervical and inguinal lymphadenopathy. By the 16th day of her rash, most VP had denuded resulting in erythematous patches that were particularly confluent on her face and trunk (Figure 2a,b). At that point, her absolute eosinophil count was 0.9 × 10^9^ cells/L and aspartate and alanine transaminases had increased to 357 and 339 U/L, respectively (Table 1). Infectious workup was unremarkable including negative testing for cytomegalovirus (CMV), COVID‐19, hepatitis A, B and C, herpes simplex virus 1 and 2, human immunodeficiency virus, human herpesvirus 6 (HHV‐6), MPox, *Mycoplasma pneumoniae*, rubella, rubeola, and varicella zoster virus. Rapid plasma reagin, antinuclear antibody, c‐reactive protein, erythrocyte sedimentation rate, and lactate dehydrogenase were similarly unremarkable. A punch biopsy of a vesicular lesion on her right upper back performed at admission revealed a very mild superficial and deep perivascular dermatitis with a focal aggregate of atypical hematolymphoid cells in the deep dermis (Figure 3a–c).

**TABLE 1:** Pertinent laboratory findings and management decisions during hospital course.

Hospital day	Rash day	Rash evolution	Absolute eosinophil count (× 10^9^ cells/L)^a^	AST (U/L)^b^	ALT (U/L)^b^	RegiSCAR score	Management
1	9	Face, trunk, and upper and lower extremities: Numerous VP with surrounding erythema	‐	‐	‐	−1	Discontinued anticonvulsants, obtained biopsy, and started supportive care
2	10	Hands: Developed new vesicles bilaterally	0.3	38	33	−1	Supportive care
3	11	Unchanged	0.4	‐	‐	−1	Supportive care
4	12	Face, trunk, and upper and lower extremities: VP beginning to denude	0.5	‐	‐	−1	Supportive care
5	13	Upper and lower extremities: Confluent erythematous patches with mostly denuded VP; erythematous plaques with overlying scale on bilateral inner thighs	0.7	65	71	2	Supportive care
6	14	Face and trunk: Confluent erythematous patches with mostly denuded VP	0.9	98	99	2	Supportive care
7	15	Unchanged	0.9	161	168	5	Supportive care
8	16	Unchanged	0.9	357	339	5	IV methylprednisolone 500 mg daily
9	17	Decreasing erythema	0	215	342	4	IV methylprednisolone 500 mg daily
10	18	‐	0	151	298	‐	IV methylprednisolone 500 mg daily
11	19	‐	0	74	234	‐	Discharged on prednisone 150 mg daily for 3 days

*Note*: At admission, RegiSCAR was −1 due to lack of fever (−1), lymphadenopathy ≥2 sites (+1), no eosinophilia (0), no atypical lymphocytes (0), rash involving >50% body surface area (+1), not having ≥2 cutaneous characteristics suggestive of DRESS (e.g., facial oedema, infiltration, psoriasiform desquamation, and/or purpura) (−1), biopsy results unknown (0), no signs of internal organ involvement (0), rash not yet present for ≥15 days (−1), and serologic testing for alternate aetiologies not yet performed (0). On day 5 of hospitalisation, RegiSCAR increased to 2 due to absolute eosinophil count falling within the range of 0.700–1.499 cells × 10^9^/L (+1) and ≥2 cutaneous characteristics suggestive of DRESS (facial oedema and psoriasiform desquamation) (+1). On day 7 of hospitalisation, RegiSCAR increased to 5 due to confirmation of hepatic involvement (ALT twice the upper limit of normal for two consecutive days) (+1), lack of resolution at rash day 15 (+1), and negative serologic evaluations for other aetiologies (+1).

Abbreviations: ALT, alanine transaminase; AST, aspartate transaminase; IV, intravenous; RegiSCAR, registry of severe cutaneous adverse reactions; VP, vesicles and pustules.

^a^ Laboratory upper limit of normal for absolute eosinophil count is 0.4 cells × 10^9^/L.

^b^ Laboratory upper limit of normal for both AST and ALT is 35 U/L.

She was diagnosed with DRESS on the 16th day of her rash. Her Registry of Severe Cutaneous Adverse Reactions (RegiSCAR) score was five. Given her drug timeline, levetiracetam was the suspected trigger although the possibility of valproate inducing DRESS could not definitively be excluded. Both anticonvulsants were added to her list of allergies, and lacosamide was started for epilepsy. Given her rapidly worsening transaminitis, treatment with intravenous methylprednisolone 500 mg daily was initiated immediately after diagnosis. Within 24 h, she had decreased pruritus, erythema, eosinophils, and transaminase levels. Intravenous methylprednisolone was administered for a total of 3 days. She was then was discharged on prednisone 150 mg daily for 3 days, followed by 90 mg daily to be tapered over 8 weeks.

## 3 DISCUSSION

While the rash of DRESS is often thought to be morbilliform, only 15% of patients with DRESS have a monomorphic morbilliform rash and the remaining 85% have polymorphic features, which can include blisters, exfoliation, purpura, pustules, and urticaria.^3^, ^4^ The co‐occurrence of pustules with a morbilliform rash may occur in 4.8%–30% of instances.^3^, ^4^ More rarely, as in our case, isolated pustules can develop without a preceding morbilliform rash, termed pustular DRESS.^5^, ^6^

Although potentially leading to a delay in diagnosis, neither pustules in isolation nor those associated with a morbilliform rash have been linked to more severe disease.^4^, ^5^, ^6^ More generally, none of the varying cutaneous findings of DRESS have been associated with differences in overall survival.^4^, ^7^ Nevertheless, significantly worse hepatic involvement has been observed in patients with an erythema multiforme‐like rash.^7^

There are no universally adopted diagnostic criteria for DRESS, but the RegiSCAR score proposed in 2007 is widely used.^2^, ^8^ Values between 4 and 5, like in our patient, indicate a probable case. Our diagnosis was further supported by levetiracetam being initiated within 2–6 weeks prior to disease onset and the exclusion of alternate aetiologies, including acute varicella, disseminated herpes simplex, Epstein‐Barr virus (EBV) infection, Kikuchi‐Fujimoto disease, and acute generalised exanthematous pustulosis (AGEP). Pustules, facial oedema, and an eosinophilia can all occur in AGEP, and patients meeting the diagnostic criteria for multiple drug reactions have been reported.^2^, ^9^ However, our case is more consistent with DRESS than AGEP given the latency period (41 vs. <4 days), disease duration (≥15 vs. <15 days), lymphadenopathy, and hepatic injury.^1^, ^2^

While our final pathology read was performed following the diagnosis and initiation of treatment, the observed perivascular inflammation with a focal cluster of atypical hematolymphoid cells may be supportive. The histopathology of DRESS is often varied and non‐specific, but perivascular lymphocytosis is commonly observed as are dyskeratosis, epidermal spongious, interface vacuolisation, and eosinophilic infiltration.^1^, ^2^, ^10^ Atypical lymphocytes are not uncommon with one study reporting this finding in 28% of cases.^11^ Neutrophils were not observed in our case and would be atypical for DRESS.^1^, ^2^ Interestingly though, the presence of neutrophils on histology have been reported in previous cases of pustular DRESS.^5^, ^6^

The exact pathophysiology of DRESS remains undetermined. However, it is considered a T‐cell‐mediated delayed hypersensitivity reaction whereby drugs and their metabolites stimulate the immune system by either serving as haptens or directly binding to human leucocyte antigens (HLA) and/or T‐cell receptors.^1^, ^12^ Viral reactivation has also been implicated, which could explain why symptoms can worsen or flare even after drug discontinuation.^12^ Herpes viruses have been observed to reactivate sequentially during the disease course beginning with HHV‐6 and/or EBV, followed by human herpesvirus 7 (HHV‐7) and CMV.^13^ Our patient's infectious workup including serologic testing for herpes viruses was negative. However, EBV was indeterminate due to the presence of polymerase chain reaction inhibitors in the sample, and we could not investigate for HHV‐7 as our lab does not have this capability.

Aromatic anticonvulsants, such as phenytoin, carbamazepine, phenobarbital, and lamotrigine, are among the most common causes of DRESS.^3^, ^4^ Of the 117 validated cases of DRESS in the prospectively collected multinational RegiSCAR registry from 2003 to 2009, over one third were caused by anticonvulsants, all belonging to the aromatic class.^3^ The newer, non‐aromatic antiepileptics, such as levetiracetam, are generally considered safer, but there has been a growing number of levetiracetam‐induced DRESS cases since 2010.^14^ Moreover, in a 2019 investigation of antiepileptics causing severe cutaneous reactions in Korea, DRESS was induced by levetiracetam in nine of 89 (10%) instances.^15^ Study authors hypothesised that this high prevalence may suggest the presence of an HLA allele in the Korean population conferring levetiracetam sensitivity. Another explanation may be the increasing use of non‐aromatic antiepileptics in recent years.

Applying the Liverpool Adverse Drug Reaction causality assessment tool, levetiracetam was a probable culprit while valproate was unlikely. Generally, for further determination of causative agents, both in vitro and ex‐vivo tests can be performed (e.g., lymphocyte transformation test, lymphocyte toxicity assay, and enzyme‐linked immunoSpot), but they have variable sensitivities, have not been validated in large‐scale studies, and are not available at our institution.^2^, ^16^ Notably, it has been asserted that drug patch testing should be the first diagnostic test used in identifying culprit drugs.^17^ Our patient was offered drug patch testing, but she declined.

## 4 CONCLUSION

DRESS has a range of cutaneous morphologies. Pustules may be present in isolation or as a secondary feature of a morbilliform rash and should not exclude this diagnosis. Similarly, while rare, it is important to consider the possibility of levetiracetam inducing severe cutaneous adverse reactions.

## CONFLICT OF INTEREST STATEMENT

Norman, Guenther, Wu, DeClerck, and Worswick report no conflicts of interest.

## AUTHOR CONTRIBUTIONS

**Thomas Norman**: Conceptualization (equal); writing—original draft (lead); writing—review and editing (equal). **Jana Guenther**: Conceptualization (supporting); writing—review and editing (equal). **Kevin Wu**: Conceptualization (equal); writing—review and editing (equal). **Brittney DeClerck**: Writing—review and editing (equal). **Scott Worswick**: Conceptualization (equal); supervision (lead); writing—review and editing (equal).

## ETHICS STATEMENT

Patient provided consent for publishing details of her case including the enclosed images.

## Data Availability

Data sharing not applicable to this article as no datasets were generated or analysed during the current study.
